# Individual Differences in the Amount and Timing of Salivary Melatonin Secretion

**DOI:** 10.1371/journal.pone.0003055

**Published:** 2008-08-26

**Authors:** Helen J. Burgess, Louis F. Fogg

**Affiliations:** Biological Rhythms Research Laboratory, Department of Behavioral Sciences, Rush University Medical Center, Chicago, Illinois, United States of America; Vanderbilt University, United States of America

## Abstract

The aim of this study was to examine individual differences in a large sample of complete melatonin profiles not suppressed by light and search for possible associations between the amount and timing of melatonin secretion and a multitude of lifestyle variables. The melatonin profiles were derived from saliva samples collected every 30 minutes in dim light from 85 healthy women and 85 healthy men aged 18–45 years. There was a large individual variability in the amount of melatonin secreted with peak values ranging from 2 to 84 pg/ml. The onset of melatonin secretion ranged from 18:13 to 00:26 hours. The use of hormonal birth control, reduced levels of employment, a smaller number of days on a fixed sleep schedule, increased day length and lower weight were associated with an increased amplitude of melatonin secretion. The use of hormonal birth control, contact lenses, a younger age, and lower ratings of mania and paranoia were associated with a longer duration of melatonin secretion. An earlier occurrence of the onset of melatonin secretion was associated with an earlier wake time, more morningness and the absence of a bed partner. Lifestyle and behavioral variables were only able to explain about 15% of the individual variability in the amount of melatonin secretion, which is likely because of a substantial genetic influence on the levels of melatonin secretion.

## Introduction

Melatonin is a hormone synthesized and released from the pineal gland [1]. The master circadian clock, located in the suprachiasmatic nuclei (SCN) in the hypothalamus, controls the secretion of melatonin from the pineal gland via a multisynaptic pathway involving the paraventricular nucleus, spinal cord and superior cervical ganglion. Typically, melatonin levels begin to increase before sleep, peak in the early hours of the morning and decrease to daytime levels after waking. Endogenous melatonin secretion is suppressed by light according to a dose response curve [2], such that melatonin can even be suppressed by ordinary indoor light [3]. The extent of melatonin suppression due to light is also dependent on prior light exposure, with greater previous light exposure resulting in less melatonin suppression [4].

The duration of melatonin secretion corresponds to the length of the dark period as a result of the suppressing effects of melatonin by light. Thus, in many animals, the duration of the melatonin profile is an important humoral signal that transmits information about day length (photoperiod) to the neuroendocrine-gonadal axis, thereby regulating appropriate seasonal physiological and behavioral changes. However in humans, the use of indoor lighting at night results in most humans experiencing a summer photoperiod year round [5]. Indeed, humans living in mid latitudes do not typically show seasonal changes in their melatonin duration despite the changing external photoperiod [6], [7]. However, when humans were exposed to extremely long (14 hour) dark periods, melatonin duration increased, indicating that the melatonin photoperiodic response in humans remains intact. Furthermore, the increase in melatonin duration was even evident when melatonin was measured in dim light, indicating that as in other animals, the human circadian system has a “memory” for its recent photoperiodic history [8].

The melatonin rhythm in humans is of interest for at least two other reasons. First, because melatonin secretion is regulated by the master circadian clock, the dim light melatonin rhythm is increasingly being used as a reliable marker of the human master circadian clock [9], which is located in the SCN. Two commonly derived circadian phase markers include the dim light melatonin onset (DLMO), the point in time when melatonin levels begin to rise in the evening and the dim light melatonin offset (DLMOff), the point in time when melatonin levels diminish in the morning [10]. The DLMO is the most reliable circadian phase marker, more reliable than the DLMOff and phase markers derived from the core body temperature rhythm [9]. Importantly, the timing of the DLMO is not necessarily dependent on the amplitude of the melatonin secretion.

There has also been increasing interest in melatonin because of its possible role in the etiology of breast cancer. Amongst numerous effects, melatonin is a potent anti-oxidant and an anti-inflammatory agent. An anti-cancer role for melatonin in breast cancer is supported by the finding that human blood rich in melatonin slows the growth of human breast cancer xenografts in rats [11]. In humans, indirect evidence includes that night workers, who may have suppressed melatonin due to their light exposure at night, have an increased risk for breast [12] and colorectal cancer. However, there have been mixed findings from prospective studies on whether or not melatonin levels in healthy women are predictive of their future cancer risk [13], [14].

The possible connection between melatonin and cancer have led to an increasing interest in behavioral and lifestyle factors beyond light exposure, which may influence melatonin concentrations, such as age, alcohol consumption, body mass index (e.g. [15], [16]) and numerous medications (e.g. [15], [17], [18]). However, many of these studies have estimated melatonin secretion from the concentration of the melatonin metabolite 6-sulfatoxymelatonin in a urine sample collected in ambient light immediately after rising. As subjects are typically instructed to void at bedtime, the entire melatonin secretion, which begins 2–3 hours prior to habitual bedtime, may not be measured. Furthermore, a single urine sample cannot provide information on the duration of melatonin secretion (DLMO to DLMOff interval) or on the timing of the DLMO. In our laboratory we regularly measure complete salivary melatonin profiles in dim light (<10 lux) to estimate the timing of the circadian clock. We also collect much behavioral and lifestyle information from our subjects. Thus the aim of this study was to examine individual differences in the amount of melatonin secreted and timing (phase) of the melatonin rhythm in a large sample of complete salivary melatonin profiles not suppressed by light, and search for possible associations between these melatonin parameters and a multitude of lifestyle variables, some not previously examined in the literature.

## Methods

### Subjects

As part of numerous studies, we have collected complete baseline salivary melatonin profiles from 170 individual subjects ([19], [20], [21], [22], [23], [24] and unpublished data). We included all available complete melatonin profiles for this analysis. The demographics of the sample are detailed in Tables 1 and 2. All subjects were nonsmokers, free of medication throughout the study, except hormonal birth control, and were in self-reported good health. None reported taking melatonin supplements. One hundred and thirty two subjects passed a urine drug screen for common drugs of abuse (the remaining 38 subjects were not tested). No subject had worked night shifts or flown across more than 2 time zones in the previous month. Daily use of sunglasses was recorded by 107 subjects from enrollment in the study to the day of the phase assessment. If subjects typically wore contact lenses they were permitted to do so during the study. The entire protocols of all of the studies conformed to the standards set by the Declaration of Helsinki and were approved by the Rush University Medical Center Institutional Review Board. All procedures were conducted with the prior adequate understanding and written consent of the subjects.

**Table 1:** Means, standard deviation and range of continuous demographic variables.

	Mean (SD)	Range
Age (y)	26.15 (6.00)	18 to 45
Weight (kg)	70.10 (13.87)	44.48 to 105.76
Height (m)	1.71 (0.11)	1.35 to 1.98
BMI (kg/m^2^)	23.89 (3.16)	16.85 to 30.31
Morningness-eveningness^a^	52.11 (9.10)	34 to 75
Sleepiness^b^	5.69 (3.16)	0 to 14
Sleep quality^c^	2.76 (1.50)	0 to 7
Baseline days^d^	11.16 (4.37)	3 to 15
Day length (h)^e^	13.16 (1.54)	9.48 to 15.20
Caffeine (50 mg doses per week)^f^	9.19 (11.34)	0 to 70
Alcohol (drinks per week)^f^	2.45 (2.96)	0 to 18

a Assessed immediately after study enrolment, from the Horne Ostberg questionnaire [70] in all 170 subjects.

b Assessed immediately after study enrolment, from the Epworth Sleepiness Scale [71] in 132 subjects. Scores of greater than 10 are suggestive of high daytime sleepiness.

c Assessed immediately after study enrolment, from the Pittsburgh Sleep Quality Index [72] in 132 subjects. A score of greater than 5 is suggestive of a sleep disorder.

d Number of days on fixed sleep schedule prior to phase assessment.

e Sunrise to sunset times in Chicago (US Naval Observatory).

f Self-reported habitual use prior to start of study.

**Table 2 pone-0003055-t002:** Sample size of categorical demographic variables.

	Number of subjects
Sex	85 men, 85 women
Hormonal birth control	16 hormonal birth control, 69 no hormonal birth control
Menstrual phase^a^	34 follicular, 33 luteal, 2 unknown
Race	104 White, 32 Asian, 23 Black, 11 Other
Ethnicity	14 Hispanic, 156 non Hispanic
Education	3 high school, 68 some college, 44 college graduate, 44 higher degree, 11 unknown
Employment	35 unemployed, 116 student and/or part time work, 19 full time work
Living condition	37 alone, 133 not alone
Bed partner	41 bed partner, 129 no bed partner
Season	40 spring, 88 summer, 20 fall, 22 winter
Eyeglasses	61 wore eyeglasses, 68 no eyeglasses, 41 unknown
Contact lenses	55 wore contacts, 74 no contacts, 41 unknown
Sunglasses	93 yes sunglasses, 14 no sunglasses, 63 unknown
Caffeine in saliva	25 had caffeine in saliva, 145 had no caffeine in saliva

a Menstrual phase is only reported for females not using hormonal birth control.

### Protocol

All subjects slept at home and were assigned a 8–9 hour sleep schedule for 3, 7, 10 or 15 days prior to the baseline phase assessment of each study, during which we collected their melatonin profiles. The sleep schedules were assigned to match each subject's sleep reported habitual sleep times. However, 11 of the 170 subjects (6.5%) were assigned sleep schedules that varied from their self reported habitual sleep schedule by more than 1 hour but less than 2 hours. During their scheduled sleep times subjects were instructed to lie in bed and try to sleep. They were not permitted to read, watch TV, listen to music or talk on the telephone at this time. To ensure compliance subjects were required to call the laboratory voice mail (time and date of call was recorded) before turning out their lights at night and at their wake time in the morning each day. Subjects also wore a wrist actigraphy monitor (Ambulatory Monitoring BMA-32, Ardsley NY or Actiwatch-L, Mini-Mitter, Bend OR) which recorded their activity every minute and was downloaded and inspected in their presence every 1–3 days. Subjects also completed daily logs noting the time and dose of any caffeinated beverages, alcohol, and medications they consumed that day.

### Phase assessment

Each subject experienced a baseline dim light phase assessment in the laboratory to determine their endogenous melatonin profile. The light in the phase assessment room was generated from 4 ceiling fixtures, each of which contained 3 fluorescent tubes (GE 32 watt 4100K F32T8-SPX41) which were behind a red filter (Rosco E-colour RS0248). The light in the adjoining bathroom was generated by 2 light fixtures, each of which contained 1 incandescent bulb (GE 90 watt Miser). All light fixtures were controlled by dimmer switches and each switch was locked to a low setting. During the phase assessment, subjects were seated in recliners in dim light (always <10 lux, at the level of the subjects' eyes, in the direction of gaze, measured at least every 4 hours with a Minolta TL-1 light meter, Ramsey, NJ) for at least 20 hours. After no more than 45 minutes in the dim light, subjects began to give a saliva sample every 30 minutes using Salivettes (Sarstedt, Newton, NC). During the session subjects were kept awake and talked amongst each other, watched a dimmed TV (<10 lux) or listened to music. Non-steriodal anti-inflammatory drugs were not permitted for at least 72 hours before the first saliva sample as these drugs have been shown to suppress melatonin [25]. Subjects did not consume any alcohol in at least the 21.5 hours prior to the collection of the first saliva sample and were breathalyzed on arrival to ensure their blood alcohol concentration was zero. Subjects did not drink caffeine for at least 6 hours before the first saliva sample. Toothpaste or mouthwash were not allowed during the phase assessments. Small caffeine-free snacks and fluids were permitted, except in the 10 minutes before each sample, and subjects were required to rinse and brush their teeth with water while remaining seated 10 minutes before each sample if they had consumed food or drink. Subjects were occasionally allowed out of the recliners to use the toilet facilities (also <10 lux) but had to remain seated in the 10 minutes before each saliva sample. After collection, the samples were centrifuged, frozen and later shipped in dry ice to Pharmasan Laboratories (Osceola, WI) to be radioimmunoassayed for melatonin. The sensitivity of the assay was 0.7 pg/ml, and intra- and interassay coefficient of variabilities were 12.1 and 13.2% respectively.

### Data Analysis

The peak value or maximum point of each melatonin profile was identified. Additionally, area under the curve (AUC) was calculated for each melatonin profile using the trapezoidal method [26]. To ensure AUC calculations were not confounded by differences in the number of saliva samples collected between studies, longer profiles were truncated to 40 samples by removing extra low daytime points as needed. Every individual melatonin profile was visually inspected to ensure the entire profile was captured by the phase assessment.

The dim light melatonin onset (DLMO) and dim light melatonin offset (DLMOff) were also calculated. For each melatonin profile, a threshold was calculated as the mean of 3 low consecutive daytime values plus twice the standard deviation of these points [27]. Each subject's DLMO was the point in time (as determined with linear interpolation) when the melatonin concentration exceeded and remained above the threshold for at least 1 hour. The DLMOff was the point in time when melatonin levels fell and remained below the threshold for at least 1 hour. The mean (±SD) threshold was 1.8±0.9 pg/ml. Seven profiles were of such irregular pattern that these circadian phase markers could not be reliably determined, and therefore DLMOs and DLMOffs were only calculated on 163 melatonin profiles.

### Statistical Analysis

The statistical analysis included four dependent variables which were all derived from the melatonin profiles: peak value, AUC, DLMO, and melatonin duration (DLMO-DLMOff). As the DLMO can be influenced by environmental light exposure [28], [29], alternative derivations of the DLMO (DLMO to bedtime interval and sunset to DLMO interval, see Table 3) were also tested, but as they did not yield unique results only the DLMO was used in the model reported here. A total of 44 independent variables were evaluated: the lifestyle and behavioural variables listed in Tables 1–3, and the 13 subscales of the Minnesota Multiphasic Personality Inventory-2 (MMPI-2) [30]. Missing values for the continuous independent variables were replaced with the group mean. The missing values of the categorical independent variables were replaced with the hot-deck method [31].

**Table 3:** Melatonin profile characteristics and sleep times.

	Mean (SD)	Range
Peak value (pg/ml)	17.64 (12.48)	2.40 to 83.60
Area under the curve (pg/ml/0.5 h)	235.68 (155.73)	43.95 to 1063.29
DLMO (time)	20:50 (1:12)	18:13 to 00:26
DLMOff (time)	08:21 (1:25)	04:54 to 12:38
DLMO to DLMOff (h)	11.52 (1.25)	7.89 to 14.70
DLMO to bedtime (h)	2.65 (1.04)	−0.30 to 5.79
Sunset to DLMO (h)	1.59 (1.68)	−1.44 to 6.92
Prestudy bedtime	00:10 (0:55)	22:02 to 03:02
Prestudy wake time	08:11 (1:01)	05:43 to 12:04
Prestudy time in dark (h)	8.01 (0.76)	6.22 to 12.88
Assigned bedtime	23:28 (0:47)	22:00 to 02:00
Assigned wake time	07:31 (0:48)	06:00 to 10:00
Assigned time in dark (h)	8.05 (0.20)	8.00 to 9.00

A multivariate analysis was planned using structural equation modeling. The analytic model used to describe these data had a multivariate melatonin response with the four dependent variables used as manifest measures of the underlying melatonin response (n = 163). Potential predictors were entered into the model one at a time, and were discarded if the model failed to fit the data, or the t-statistic associated with the most recently entered predictor was below 1.50. Overall model fit was determined when the relative chi-square (chi-square divided by df) was below 2.00 and the root mean square error of approximation (RMSEA) was below 0.05. The final model selected had structural equation coefficients with one-tailed probabilities less than 0.05. If the model was significant, univariate stepwise regressions were planned to further explore each dependent variable. Statistical significance was determined at p<0.05.

## Results

**Figure 1:**
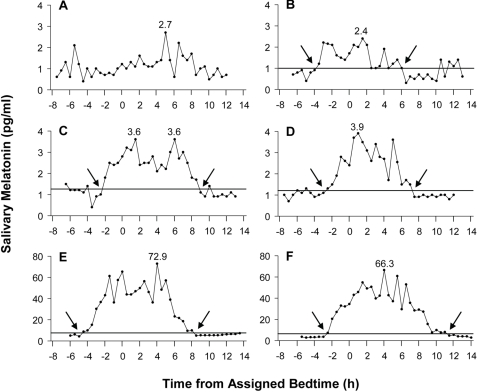
Six individual melatonin profiles determined from half hourly saliva samples collected in dim light. Shown are two melatonin profiles with the lowest AUC (A, B), two melatonin profiles with low AUC but normally shaped melatonin profiles (C, D), and two melatonin profiles with the highest AUC (E, F). The profiles are plotted with reference to assigned bedtime. The peak value of each profile is shown above the corresponding point on the profile. The threshold used to determine the DLMO and DLMOff is represented by the horizontal line. The DLMO and DLMOff are indicated by arrows.

**Figure 2:**
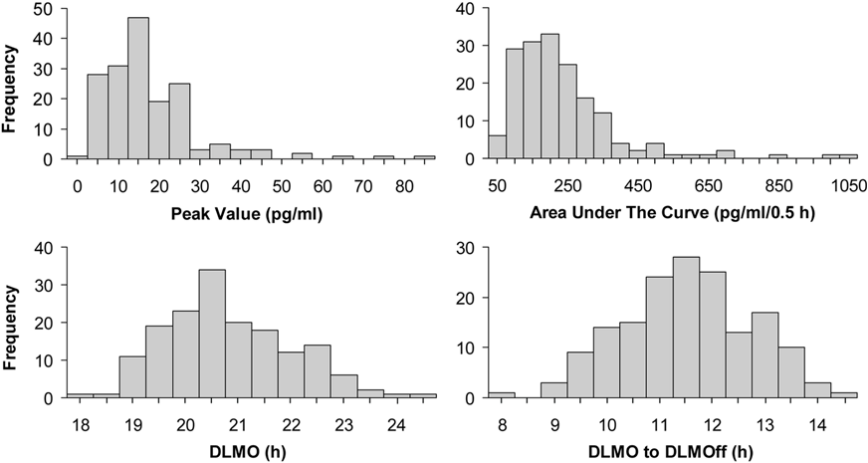
The frequency distributions of peak value, area under the curve, time of the DLMO and melatonin duration (DLMO-DLMOff). The frequency distributions of peak value and area under the curve were positively skewed, while DLMO and DLMO-DLMOff were normally distributed.

Figure 1 illustrates the variability in the individual melatonin profiles collected and that some low secretors had a normally shaped melatonin rhythm while other low secretors did not. Using our method, we were able to calculate DLMOs and DLMOffs for the low secretors in B, C and D, but not for the low secretor in A. Figure 2 illustrates the positively skewed distributions observed in the peak value and AUC, and the normal distributions observed in the DLMO and DLMO-DLMOff duration. For statistical analyses, peak value and AUC were normalized with a natural log transformation, although the means of peak value and AUC reported in the text and figures are of the untransformed variables. The mean, standard deviation and range of the peak value, AUC, DLMO, DLMOff and DLMO to DLMOff duration are shown in Table 3. AUC and peak value were significantly and highly correlated (r = 0.96, p<0.001). Peak value and DLMO-DLMOff duration were significantly correlated (r = 0.35, p<0.001) as was AUC and DLMO-DLMOff duration (r = 0.46, p<0.001). There was no significant correlation between DLMO and peak value or AUC (both r≤0.11, p≥0.15).

The final structural equation model fit the data well (chi-square = 25.35, df = 26, relative chi-square = 0.98, RMSEA = 0.001). There were four significant predictors of melatonin response in the model: age (r = .18), a dummy-coded measure of level of employment (r = 0.14), a dummy-coded measure of use of birth control pills (r = −.18) and two scales from the MMPI-2, paranoia (r = .17) and hypomania (r = .17). The results of the univariate stepwise regressions (n = 170 for peak value and AUC, n = 163 for DLMO and DLMO-DLMOff) are described below. The regressions were only able to explain 14.8, 13.7, 35.7 and 14.6% of the variance in the peak value, AUC, DLMO and DLMO-DLMOff duration respectively.

### Age, Sex, Hormonal Birth Control, Menstrual Phase

**Figure 3:**
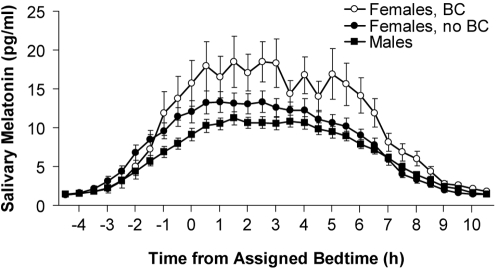
The mean melatonin profiles of females using (“BC”, n = 16) or not using hormonal birth control (“no BC”, n = 69) and males (n = 85). The profiles are plotted with reference to assigned bedtime. Error bars represent SEMs.

Increasing age was associated with a shorter DLMO-DLMOff duration (B = −0.04, p<0.03), but not with any of the other melatonin parameters. For every 10 year increase in age, the DLMO-DLMOff duration decreased by 0.36 hours. Sex and menstrual phase were not significantly associated with any of the melatonin parameters. Use of hormonal birth control was associated with a higher peak value (B = 0.41, p<0.02), higher AUC (B = 0.37, p<0.02) and a longer DLMO-DLMOff duration (B = 0.64, p<0.05). Figure 3 shows the mean melatonin profiles of females who used hormonal birth control, females who did not use hormonal birth control and males.

### Weight, Height, Body Mass Index (BMI)

Increased weight was associated with a lower peak value (B = −0.008, p<0.05) and a lower AUC (B = −0.006, p<0.05), such that an additional 10 kgs (about 22 lbs) corresponded to a reduction in peak value of 9.93 pg/ml and reduction in AUC of 9.94 pg/ml/0.5 h. Height and BMI were not associated with any of the melatonin parameters.

### Race, Ethnicity, Education, Employment, Living Condition, Bed Partner

**Figure 4:**
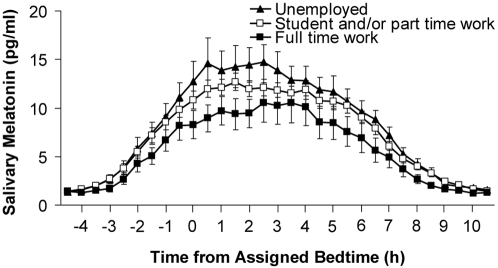
The mean melatonin profiles of the unemployed (n = 35), students and/or part-time workers (n = 116) and full-time workers (n = 19). The profiles are plotted with reference to assigned bedtime. Error bars represent SEMs.

Race, ethnicity (NIH classification), education level and living condition were not associated with any melatonin parameter. However there were effects of employment status. For this analysis students and part-time workers were grouped together because many students also worked part-time. Increased levels of employment were associated with reduced AUC (B = −0.17, p = 0.03). Figure 4 shows the mean melatonin profiles of the unemployed, student/part-time workers and full-time workers. Having a bed partner was associated with a later DLMO (B = −0.37, p<0.05), on average a 0.37 hour delay in the DLMO.

### Morningness-Eveningness, Sleepiness, Sleep Quality, Personality

Table 1 shows the mean morningness-eveningness, sleepiness and sleep quality scores. These were all assessed immediately after study enrollment and thus prior to the phase assessment. More morningness (higher score) was associated with an earlier DLMO (B = −0.03, p<0.01). Sleepiness (from the Epworth Sleepiness Scale) and sleep quality (from the Pittsburgh Sleep Quality Index) were not associated with any melatonin parameter. None of the validity scales (L, F, K) or the clinical scales of Hypochodriasis, Depression, Hysteria, Psychopathic Deviate, Masculinity-Femininity, Psychasthenia, Schizophrenia and Social Introversion on the Minnesota Multiphasic Personality Inventory-2 (MMPI-2) [30] were associated with any melatonin parameter. However, increased scores of Hypomania and Paranoia were associated with a shorter DLMO-DLMOff interval (B = −0.02, p<0.05 and B = −0.02, p = 0.05 respectively).

### Sleep Times, Number of Baseline Days, Day Length, Season

Mean sleep times are shown in Table 3. Prestudy sleep times were not associated with any melatonin parameter. An earlier assigned wake time was associated with an earlier DLMO (B = 0.77, p<0.001), such that for each earlier hour of scheduled wake time, the DLMO was advanced by 0.77 hours. A greater number of days on the assigned sleep schedule was associated with a lower peak value (B = −0.04, p<0.001), and a lower AUC (B = −0.03, p<0.01), such that for each additional day of scheduled sleep, peak value decreased by 0.96 pg/ml and AUC by 0.97 pg/ml/0.5 h. The summary statistics of day length (sunrise to sunset) are shown in Table 1. Increased day length was associated with a higher peak value (B = 0.11, p<0.01) and a higher AUC (B = 0.07, p<0.05). For every additional hour of day length, peak value and AUC increased by 1.12 pg/ml and 1.07 pg/ml/0.5 h respectively. There were no effects of season on any of the melatonin parameters.

### Eyeglasses, Contact Lenses, Sunglasses

There was no effect of eyeglasses or sunglasses use on any melatonin parameter. However, the use of contact lenses was associated with a longer DLMO-DLMOff duration (B = −0.39, p<0.05), such that subjects who wore contact lenses had an average 0.39 hour increase in melatonin duration.

### Caffeine, Alcohol

Habitual caffeine and alcohol use was determined from self-report prior to the start of each study and summary statistics of these variables are in Table 1. Neither habitual caffeine use or alcohol use were significantly associated with any melatonin parameter. Using the daily logs subjects completed during the studies, we identified 25 subjects who could have had circulating levels of caffeine during the saliva collection. However there was no association between circulating levels of caffeine and any of the melatonin parameters.

## Discussion

In this study, 170 salivary melatonin profiles that were sampled half hourly and not suppressed by light were examined. To our knowledge this is the largest sample of its kind reported. There was a large individual variability and a positively skewed distribution in the amplitude of melatonin secreted (peak value, AUC, Figures 1, 2). Thus it is more unusual to secrete very high amounts of melatonin than to secrete very low amounts of melatonin (e.g.[15], [32], [33]). By contrast, the distributions of the DLMO and DLMO-DLMOff duration were normally distributed. Previous research in sheep suggests that the variability in the amount of melatonin secreted is due to differences in the synthesis, rather than metabolism, of melatonin [34]. The amount of melatonin secreted could be due to a multitude of factors, including the number of functioning (uncalcified) pinealocytes in the pineal gland [35], [36] and the integrity of the retinal-suprachiasmatic nuclei-pineal pathway [37].

The relationships between melatonin secretion and numerous behavioral and lifestyle variables were also examined. As detailed below, several variables had significant associations with the amount and timing of melatonin secretion; however all of these variables combined were only able to explain about 15% of the individual variability in the amount of melatonin (peak value, AUC, DLMO-DLMOff), but 36% of the individual variability in the DLMO. Thus all of these significant variables are likely modifying a strong genetic influence on the amount of melatonin secretion as indicated by high correlations in the plasma melatonin levels in monozygotic twins (r = 0.94, [38]). Indeed, individual melatonin profiles often have a characteristic shape that is reproducible with repeated assessment (e.g. Figure 3 in [20], Figure 2 in [39]). Below the specific findings and possible mechanisms are discussed and related to the existing literature.

There was a small effect of age on the duration of melatonin secretion, but not on the amplitude, which may have been due to the restricted age range in this study (18–45 years). Many studies have reported decreasing melatonin levels with increasing age, but melatonin levels in these studies may have been confounded by subjects being exposed to ambient light during melatonin collection, or smoking, drinking alcohol and/or using medication in close proximity to melatonin collection (e.g. [15], [16], [17], [32], [40], [41], [42]). The one study that sampled melatonin frequently in dim light from healthy drug free subjects with ages ranging from 18 to 81 years, did not find any association between the AUC of melatonin secreted and age [33].

There was no effect of sex on any of the melatonin parameters. Furthermore, menstrual phase in women not using hormonal birth control was not associated with alterations in any of the melatonin parameters, in agreement with previous studies [39], [43], [44], [45]. Use of hormonal birth control was significantly associated with increased melatonin secretion, a result which is at odds with some epidemiological studies that measured 6-sulfatoxymelatonin [16], [18], but consistent with laboratory studies that measured melatonin in saliva or plasma [45], [46]. While the mechanism is not clear, this finding is also consistent with estradiol increasing melatonin synthesis in rat and guinea pig pineal explants [47]. Thus a previous report of an apparent sex difference in melatonin secretion, with females secreting more melatonin than males [32], could be because some females in the sample were using hormonal birth control. Our sample included two female subjects using non-pill hormonal birth control (implant and vaginal ring), indicating that only inquiring about oral contraceptive use may overlook some users of hormonal birth control.

There was an association between weight and melatonin such that people of heavier weight secreted less melatonin (peak value, AUC). This finding is consistent with reports of increasing weight [32] and higher BMI [15], [16], [17] being associated with lower amounts of 6-sulfatoxymelatonin in urine. However the mechanism behind this association has not been clearly explained in the literature [17]. There were no effects of race, ethnicity, education, or living condition on melatonin. One study has previously reported increased melatonin secretion in people with higher education, even after controlling for BMI, alcohol, medication use and smoking [17]. However while 53% of that sample had received some college education, over 90% of subjects in our sample had received some college education. Thus effects of education on melatonin levels may have been obscured in our sample. Having a bed partner was found to be significantly associated with a later DLMO, although the effect was relatively small.

To our knowledge, the effect of employment status on melatonin levels has not been previously examined. As the level of employment increased, the amount of melatonin secreted, as indexed by the AUC, decreased (Figure 4). It is not entirely clear why employment would be associated with AUC but not peak value. Although the full time workers had earlier prestudy (habitual) bedtime and wake times than students/part-time workers and the unemployed (on average at least 0.35 hour earlier), their prestudy time in dark and scheduled sleep times and time in dark did not differ. This may explain why there was no effect of employment on the DLMO-DLMOff duration.

There was no significant association between the amount of melatonin secreted and morningness-eveningness score, which is consistent with a previous report [18]. However, as expected, more morningness was associated with an earlier DLMO. Baseline sleepiness and sleep quality were not associated with any melatonin parameter. This is in agreement with previous reports of no association between sleep architecture and salivary or plasma melatonin levels in young healthy subjects [48], [49] and no association between the Pittsburgh Sleep Quality Index and melatonin levels in older healthy subjects [50]. Interestingly, increased scores of hypomania and paranoia were associated with a reduced DLMO-DLMOff duration. As individuals with a hypomania T score >75 (apart from one subject with a T score of 85) and a paranoia T score ≥75 were excluded from the study, subjects who scored at higher levels on these subscales likely only had a tendency towards over activity and a paranoid predisposition [51]. Precisely why these two subscales would relate to melatonin duration is unclear. To our knowledge the only other psychological factor associated with melatonin duration is winter depression. The relationship is in the same direction, but at the other extreme: winter depression co-occurs with an increased duration in melatonin [52].

Sleep lengths and season were not significantly associated with any of the melatonin parameters. An earlier scheduled wake time was associated with an earlier DLMO, which is consistent with a previous study which manipulated wake time and found corresponding shifts in the DLMO [53]. All other sleep times were not associated with any melatonin parameter. An increasing number of days on the assigned sleep schedule was associated with reductions in melatonin amplitude. While the cause of this effect is unknown, there was a 11.52 pg/ml difference in peak value and 11.64 pg/ml/0.5 h difference in AUC between those subjects on a fixed sleep schedule for 3 versus 15 days. Consistent with previous studies of people at mid latitudes, there was no association between day length and melatonin duration [6], [7]. But longer day lengths were associated with higher peak and AUC levels of melatonin. Studies conducted at mid latitudes have either not found an effect of day length on melatonin amplitude [16], [17], [54], or found that melatonin amplitude decreases with longer day lengths [15]. One possible explanation for our results is that longer day lengths in Chicago likely leads to longer periods of time spent outside and more exposure to bright outdoor light during the day. Two research groups have reported that more bright light during the day can increase melatonin amplitude at night [55], [56]. Further research is needed to fully investigate this possibility.

To our knowledge, this is the first report to explore the relationship between melatonin levels and use of eyeglasses and contact lenses. Use of eyeglasses and sunglasses were not associated with any melatonin parameter. However, use of contact lenses was associated with an on average 0.39 hour increase in melatonin duration. In humans, melatonin suppression occurs in response to short wavelength light as low as 420 nm [57], [58], but shorter wavelengths have not been tested, presumably for safety reasons. Studies which have used glasses that block light below 530–540 nm report less melatonin suppression in response to light [59], [60]. As contact lenses typically contain a UV filter, which blocks wavelengths of less than ∼400 nm [61], use of contact lens may reduce exposure to some short wavelength light. By contrast the use of eyeglasses still enables at least 7% of light to reach the eye without passing through the lens [62], presumably from the periphery. Thus it is possible that our subjects who wore contact lens received less short wavelength light prior to and during the saliva collection. A history of less melatonin suppression may have been reflected in the dim light melatonin profiles collected, or alternatively less melatonin may have been suppressed during the saliva collection. Regardless, this finding requires replication and further investigation.

There was no significant association between self-reported habitual caffeine use and the timing or amount of melatonin secreted. This is consistent with a study that reported no difference in 6-sulfatoxymelatonin levels between subjects who were high and low caffeine users [17]. Caffeine has a long half-life which ranges between 2.5 to 10 hours across individuals [63]. On average it takes 16 hours before a 100 mg dose of caffeine is eliminated and about 29 hours for a 200 mg dose to be eliminated [63]. Nonetheless, there were no differences in any of the melatonin parameters between the 25 subjects that likely had circulating levels of caffeine during the saliva collection and those that did not. Indeed, there are mixed findings in terms of the effect of caffeine on melatonin levels [64], [65], [66], [67]. Thus whatever the effects of caffeine on melatonin, the effect is likely small and the results suggest that low to moderate caffeine use does not appear to have any long term effects on melatonin. Alcohol ingestion just prior to melatonin collection has been shown to decrease melatonin [68]. However, in our study subjects did not drink alcohol for at least 21.5 hours prior to their saliva collection and were breathalyzed just prior to their first saliva sample to ensure they had zero blood alcohol concentration. Thus there were no significant associations between self-reported habitual alcohol use and the timing or amount of melatonin secretion.

We report these results in the hope that they may aid future research studies which examine the magnitude and timing of the melatonin rhythm. There are several factors which may have increased the variability in the melatonin measurement. It has recently been reported that the sleep deprivation associated with overnight phase assessments can reduce melatonin amplitude by 6.7% [69]. This would suggest that the melatonin amplitudes reported here may be slightly suppressed. Variability in the melatonin assay would also have contributed to variability in the melatonin results. However, due to the typically rapid rise in melatonin levels, assay variability likely had a greater effect on the estimates of AUC and peak value, than on the DLMO, DLMOff and melatonin duration. Despite these sources of variability, the relationships between melatonin and the various lifestyle factors examined here are not expected to have been systematically affected.

In summary, the use of hormonal birth control, reduced levels of employment, a smaller number of days on a fixed sleep schedule, increased day length and lower weight were associated with an increased amount of melatonin secretion. The use of hormonal birth control, contact lenses, a younger age, and lower ratings of mania and paranoia were associated with a longer duration of melatonin secretion. An earlier occurrence of the onset of melatonin secretion was associated with an earlier wake time, more morningness and the absence of a bed partner. About 75% of the large individual variability in the amount of melatonin secreted could not be explained, likely because of the large genetic influence on levels of melatonin secretion. Importantly, high levels of melatonin do not appear to be necessary to communicate photoperiodic information to the organism–the duration of melatonin secretion is likely the critical signal. The melatonin rhythm as a circadian phase marker is also not affected by the amplitude of melatonin secretion. Some low secretors failed to show a readily discernible circadian rhythm in melatonin secretion (Figure 1 A), but other low secretors exhibited a clear circadian pattern in their melatonin secretion (Figure 1 C, D). As levels of melatonin in the blood are approximately three times higher than in saliva, further detailed examination of low secretors may benefit by examining plasma melatonin profiles collected in dim light. Whether low melatonin secretors are at greater risk for breast cancer is currently unclear [13], [14].
